# Clinical and epidemiological factors associated with spontaneous preterm birth: a multicentre cohort of low risk nulliparous women

**DOI:** 10.1038/s41598-020-57810-4

**Published:** 2020-01-21

**Authors:** Renato T. Souza, Maria L. Costa, Jussara Mayrink, Francisco E. Feitosa, Edilberto A. Rocha Filho, Débora F. Leite, Janete Vettorazzi, Iracema M. Calderon, Maria H. Sousa, Renato Passini, Philip N. Baker, Louise Kenny, Jose G. Cecatti, The Preterm SAMBA study group, Mary A. Parpinelli, Karayna G. Fernandes, Rafael B. Galvão, José Paulo Guida, Danielly S. Santana, Kleber G. Franchini, Bianca F. Cassettari, Lucia Pfitscher, Luiza Brust, Elias F. Melo, Danilo Anacleto, Daisy de Lucena, Benedita Sousa

**Affiliations:** 1 Department of Obstetrics and Gynaecology, University of Campinas (UNICAMP) School of Medical Sciences, Campinas, SP Brazil; 2 MEAC – School Maternity of the Federal University of Ceará, Fortaleza, CE Brazil; 3 Department of Maternal and Child Health, Clinics Hospital, Federal University of Pernambuco, Recife, PE Brazil; 4 Department of Obstetrics and Gynaecology, Maternity of the Clinics Hospital, Federal University of RS, Porto Alegre, RS Brazil; 5 Department of Obstetrics and Gynaecology, Botucatu Medical School, Unesp, Botucatu, SP Brazil; 6 Statistics Unit, Jundiai School of Medicine, Jundiaí, SP Brazil; 7 College of Life Sciences, University of Leicester, Leicester, United Kingdom; 8 Faculty of Health and Life Sciences, University of Liverpool, Liverpool, UK; 9 LNBio, Campinas, Brazil

**Keywords:** Health care, Risk factors

## Abstract

The objective of this study was to determine incidence and risk factors associated with spontaneous preterm birth (sPTB). It was a prospective multicentre cohort study performed in five Brazilian referral maternity hospitals and enrolling nulliparous women at 19–21 weeks. Comprehensive maternal data collected during three study visits were addressed as potentially associated factors for sPTB. Bivariate and multivariate analysis estimated risk ratios. The main outcomes measures were birth before 37 weeks due to spontaneous preterm labour or premature rupture of membranes (sPTB). The comparison group was comprised of women with term births (≥37weeks). Outcome data was available for 1,165 women, 6.7% of whom had sPTB, 16% had consumed alcohol and 5% had used other illicit drugs during the first half of pregnancy. Current drinking at 19–21 weeks (RR 3.96 95% CI [1.04–15.05]) and a short cervix from 18–24 weeks (RR 4.52 95% CI [1.08–19.01]) correlated with sPTB on bivariate analysis. Increased incidence of sPTB occurred in underweight women gaining weight below quartile 1 (14.8%), obese women gaining weight above quartile 3 (14.3%), women with a short cervix (<25 mm) at 18–24 weeks (31.2%) and those with a short cervix and vaginal bleeding in the first half of pregnancy (40%). Cervical length (RR_adj_ 4.52 95% CI [1.08–19.01]) was independently associated with sPTB. In conclusion, the incidence of sPTB increased in some maternal phenotypes, representing potential groups of interest, the focus of preventive strategies. Similarly, nulliparous women with a short cervix in the second trimester require further exploration.

## Introduction

Spontaneous preterm birth (sPTB) is a common major pregnancy complication leading to perinatal morbidity and mortality as well as short- and long-term sequelae^1–3^. Around two-thirds of deliveries that occur before 37 weeks are due to spontaneous preterm labour or preterm premature rupture of membranes (pPROM) and its pathophysiology remains unclear^3^. The two main known risk factors for preterm birth (PTB) are a previous history of preterm birth and multiple pregnancies^1,3,4^. These factors are highly associated with preterm birth, although they cannot be applied to all women, such as nulliparous with singleton pregnancies.

Several biophysical, biological and clinical markers have been studied to identify women at high risk in a timely manner. Assessment is aimed at providing a more specialized prenatal care in referral centres capable of optimizing preventive interventions such as progesterone, pessary or cerclage^5–8^. Women with a short cervix in the second trimester, defined as a cervical length ≤25 mm measured by transvaginal ultrasound between 18 to 24 weeks of gestation^9^, are four to five times more likely to have a spontaneous preterm birth than women with a normal cervix (>25 mm)^10,11^. Nevertheless, cervical length (CL) seems to vary according to ethnicity and parity^10,12^, and possibly has a different impact on distinct populations.

Routine universal screening of cervical length remains controversial and it is not widely recommended^13,14^. Nevertheless, determining its association with sPTB in different populations may be significant for a better investigation of appropriate preventive interventions in targeted subgroups of women at higher risk. Since sPTB is a multifactorial complex disease, other clinical risk factors for sPTB also remain controversial; initial or pre-pregnancy body mass index (BMI), weight gain during pregnancy and sociodemographic factors are lacking in consistency^15–17^.

Therefore, our aim is to assess the incidence of clinical or epidemiological risk factors associated with sPTB in nulliparous women in Brazil. The determination of risk factors is an important strategy to identify women at higher risk for this important complication which represents a great burden for maternal and perinatal health. Earlier identification of women at increased risk is crucial for implementation of preventive strategies and planning adequate obstetric care.

## Methods

We conducted a longitudinal multicentre cohort, the Preterm-SAMBA study, from July 2015 to July 2018 in five Brazilian obstetric centres located in three regions of Brazil. The research protocol and other methodological details were previously published^18–21^. Briefly, the study was developed to identify clinical and biological predictors of sPTB, applying metabolomics techniques in maternal blood samples. The study protocol was approved by the Institutional Review Board (IRB) of each centre and endorsed by the Brazilian National Committee for Ethics in Research (CONEP). This manuscript follows the Strengthening the Reporting of Observational studies in Epidemiology (STROBE) Statement.

### Participants and settings

**Figure 1:**
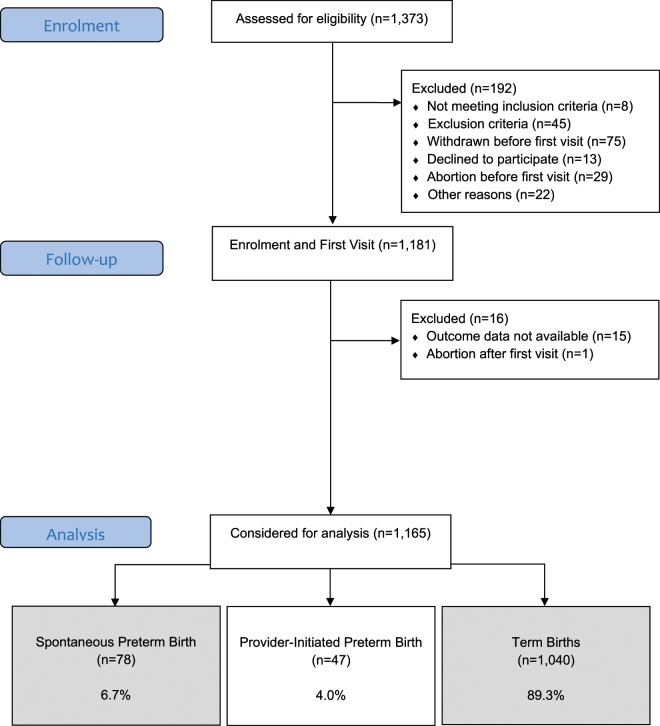
Preterm SAMBA Flowchart – Spontaneous preterm birth analysis.

Inclusion criteria included nulliparous women (no previous delivery >20 weeks), singleton pregnancy and gestational age up to 21 weeks of gestation. Exclusion criteria included 3 or more previous abortions; cervical suture; fetal malformation; chronic hypertension requiring antihypertensive drugs, diabetes or renal disease; arterial blood pressure above 160 × 100 mmHg on enrolment; Systemic Lupus Erythematosus or antiphospholipid syndrome; sickle cell disease; HIV infection; Müllerian anomalies; history of cervical knife cone biopsy; chronic use of corticosteroids, aspirin, calcium, fish oil, vitamin C, vitamin E or heparin. Participating centres were five referral maternity hospitals from the Brazilian Network for Studies on Reproductive and Perinatal Health from the northeastern, southern and southeastern regions of Brazil. Nevertheless, eligible women underwent surveillance not only in maternity outpatient clinics but also in neighbouring primary healthcare units and private clinics. The reasons (concurrent) why women met exclusion criteria were: 12 women with essential hypertension treated pre-pregnancy; 5 women with pre-pregnancy diabetes; 2 women with renal disease; 8 women with lupus; 1 woman with anti-phospholipid antibody syndrome; 1 woman with sickle cell disease; 3 women with HIV/Hepatitis B or C; 2 women with uterine anomaly; 2 women who had cerclage in the current pregnancy; 1 woman with previous cold knife cone; 7 women who were taking low-dose aspirin (≤150 mg/day); 3 women who were taking heparin/low-molecular-weight heparin; 4 women who were taking calcium (>1 g/day); 4 women who were taking eicosapentanoic acid supplementation (fish oil) (Fig. 1).

### Sample size estimation

The sample size for the Preterm SAMBA cohort was calculated taking into account the primary outcome - spontaneous preterm birth. Assuming a type I error of 5% and accuracy for the test by the area under the ROC curve of at least 0.68, and to test the hypotheses with adequate power (80% of power, β = 0.2), the required sample size should be near 80 sPTB cases. The expected minimum prevalence of this outcome in Brazil is 7%, resulting in a sample size calculation of 1,150 women for the cohort. For the current analysis, we calculated the samples size for statistical power (ad-hoc) considering a prevalence of sPTB of 7.7%, odds ratio of 2.00 for smoking^22^, α = 0.05 e β = 0. 20, non-exposed/exposed = 4:1, which resulted in n1 = 892 and n2 = 223.

### Procedures and data management

Procedures for identifying eligible women, enrolling, and collecting data in the Preterm SAMBA cohort have been previously described, including methodological details of measurements^18^. Briefly, data were collected on three study visits. During the first visit, at 19–21 weeks of gestation, blood and hair samples were collected and stored appropriately for metabolomic assay and other possible future measurements. Comprehensive information on sociodemographic characteristics, reproductive family history, current or previous diseases, life-style and habits, early and late pregnancy complications and delivery and postpartum information were collected. All collected data was entered in an online database, which had hierarchical access and complete audit trail (MedSciNet®). Anthropometric measurements of maternal height, weight, body mass index (BMI), head circumference, maternal skinfolds plus a nutritional assessment based on a 24-hour diet recall were performed according to standardized methods described in the SOP. Between 27–29 weeks and between 37–39 weeks, women underwent the same evaluation, except for blood and sample collection performed only on the first visit. Late pregnancy and postpartum data were collected through medical records and prenatal chart review, or alternatively by personal interviews or phone calls in cases where childbirth occurred outside the five hospitals.

### Outcome and variables

Spontaneous preterm birth was the main outcome, defined as any delivery before 37 weeks of gestation due to spontaneous onset of labour or preterm premature rupture of membranes (pPROM). Gestational age (GA) was estimated by the last menstrual period (LMP) and/or ultrasound (US) before 20 weeks of gestation. If discordance between LMP and US was below 7 days, GA was estimated by LMP. US was the preferred method for GA estimation when discordance was above 7 days. The comparison group was comprised of women who had term birth, defined as any delivery ≥ 37 weeks of gestation.

Potential factors associated with sPTB included sociodemographic data, smoking, alcohol (self-reported and recorded if drinking or not: 3 months before pregnancy and during pregnancy until the first visit) and drug use (marijuana, cocaine, etc.; reported and recorded similarly to alcohol consumption), history of preterm birth of study participant’s mother or sisters, sPTB or low birth weight reported by study participant, body mass index on the first visit, weight gain per week between the first and second visits, cervical length recorded by transvaginal ultrasound between 18 and 24 weeks of gestation, maternal conditions (self-reported anemia, depression or chronic hypertension), complications and infections during pregnancy, including asymptomatic bacteriuria and vaginal bleeding before the first visit (until 19–21 weeks). Maternal clinical phenotype was defined by the association with some maternal characteristics including maternal BMI, family income, weight gain, vaginal bleeding, urinary infection, short cervical length (<25 mm, measured at 18–24 weeks of gestation)^9^, ethnicity and schooling level. The proportion of women in each quartile (below Q1, Q1-Q2, Q2-Q3 and above Q3) and percentile category (<p10, p10-p90, and >p90) of weight gain per week between the first and second visits were also addressed for both preterm and term groups.

### Statistical analysis

We calculated the general incidence of sPTB in the cohort and each gestational category according to the severity of prematurity: late sPTB (34–36 weeks), moderate sPTB (32–33 weeks), very sPTB (28–32 weeks) and extreme sPTB (<28 weeks). Bivariate analysis was performed to assess potential factors associated with sPTB, calculating risk ratios (RR) and 95% confidence interval (CI). The incidence of sPTB and respective 95% CI were calculated for each clinical phenotype. We conducted a Poisson logistic regression analysis to identify factors independently associated with sPTB using adjusted RR and 95% CI. Taylor series linear approximation method were used to calculate 95% CI. Only variables with a p-value < 0.25 entered the multivariate analysis model. Statistical analysis was performed using Stata v. 7.0 (StataCorp) and SPSS v. 20.0 (IBM). All analyses were adjusted for the primary sampling unit (PSU) considering the heterogeneity of the five participating centres.

### Ethical approval

This is a study derived from the Preterm SAMBA study which obtained ethical approval from the National Committee for Ethics in Research of Brazil (CONEP) and the Committee of Research Ethics (CRE) of the coordinating center, the University of Campinas (Letter of approval 1.048.565 issued on 28^th^ April 2015), and all other Brazilian participating centers: CRE from the Maternidade Escola Assis Chateaubriand of the Federal University of Ceara in Fortaleza, CRE from the Center for Health Sciences of the Federal University of Pernambuco in Recife, CRE from the Clinics Hospital of the Federal University of Rio Grande do Sul in Porto Alegre, and the CRE from the Clinics Hospital of Botucatu Medical School at the University of the State of Sao Paulo (Unesp). Each woman signed an informed consent form before enrolment in the study. The approval for the study included administrative permissions for the research team to access the data used for this analysis. The study complies with national and international regulations for experiments in human beings, including resolution CNS 466/12 of the Brazilian National Heath Council and the 1989 Declaration of Helsinki.

## Results

In total, 1,373 nulliparous pregnant women were assessed for eligibility. Of these, 1,181 were included in the Preterm SAMBA study and had the first visit (Fig. 1). Outcome data were available for 1,165 participants, 78 of whom had a spontaneous preterm birth (6.7%) and 1,040 had a term birth (89.3%). The overall preterm birth incidence, which included provider-initiated preterm birth (pi-PTB) and sPTB, was 10.7% (Supplementary S1). The incidence of sPTB in the Northeastern and Southern/Southeastern centres was 6.0% and 7.3%, respectively, although this difference was not statistically different (p-value 0.387). The proportions of late, moderate, very and extreme sPTB were 70.5%, 12.8%, 10.3% and 6.4%, respectively (Supplementary S1). In around 98% of pregnancies, gestational age was estimated or confirmed before 20 weeks by ultrasound (Supplementary S2).

Table 1 shows that none of the assessed maternal socio-demographic characteristics was associated with sPTB. The majority of participating women were aged 20–34 years (69.2% sPTB women; 67.8% term birth women), non–white (59% sPTB; 60% term birth), had less than 12 years of schooling (66.7% sPTB; 67.9% term birth) and their annual family income was 3,000 to 12,000 US$ or more. Adolescents comprised 23.1% and 25.9% in sPTB and term births groups, respectively. More than 85% of participating women received prenatal care exclusively from the public health care system.

**Table 1:** Unadjusted risks for sPTB according to some socio-demographic characteristics.

Characteristics	sPTB	Term Births	RR [95%CI]
**Region**			
Northeast	34 (43.6%)	506 (48.7%)	Ref.
South and Southeast	44 (56.4%)	534 (51.3%)	1.21 [0.69–2.12]
**Maternal age (years)**			
≤19	18 (23.1%)	269 (25.9%)	0.88 [0.55–1.42]
20–34	54 (69.2%)	705 (67.8%)	Ref.
≥35	6 (7.7%)	66 (6.3%)	1.17 [0.27–5.15]
**Ethnicity**			
White	32 (41.0%)	416 (40.0%)	Ref.
Non-white	46 (59%.0)	624 (60.0%)	0.96 [0.40–2.30]
**Marital status**			
With partner	52 (66.7%)	762 (73.3%)	Ref.
Without partner	26 (33.3%)	278 (26.7%)	1.34 [0.99–1,81]
**Maternal Occupation**			
Paid work	41 (52.6%)	512 (49.2%)	1.11 [0.74–1.66]
Housewife	13 (16.7%)	192 (18.5%)	0.95 [0.36–2.54]
Not working*	24 (30.7%)	360 (32.2%)	Ref.
**Schooling (years)**			
<12	52 (66.7%)	706 (67.9%)	Ref.
≥12	26 (33.3%)	334 (32.1%)	1.05 [0.46–2.39]
**Annual Family Income (US$)**			
Up to 3,000	2 (2.6%)	48 (4.6%)	0.53 [0.21–1.32]
3,000 to 12,000	41 (52.5%)	563 (54.1%)	0.90 [0.55–1.47]
Above 12,000	35 (44.9%)	429 (41.3%)	Ref.
**Source of prenatal care**			
Entirely public	67 (85.9%)	899 (86.4%)	0.96 [0.54–1.70]
Private/insurance/mixed	11 (14.1%)	141 (13.6%)	Ref.
**Total**	**78**	**1,040**	

*Students, unemployed and licensed.

Table 2 shows that compared to women who did not drink, current drinker were at higher risk of spontaneous preterm birth in unadjusted models (RR 3.96, 95% CI [1.04–15.05]). On the first visit, 6.4% and 7.5% of women who had sPTB and term births, respectively, were current smokers or had ceased smoking during the first half of pregnancy. Around 16% of participants consumed alcohol in the first half of pregnancy and approximately 5% had used illegal drugs. Table 3 shows that 41.1% and 43.1% of women were overweight or obese in sPTB and term birth groups, respectively. Cervical length below 25 mm from 18 to 24 weeks was associated with a higher incidence of sPTB (RR 4.52, 95% CI [1.08–19.01]). However, the mean cervical length of women who had sPTB (33.1 mm ± 9.96) or term birth (36.9 mm ± 6.35) was not statistically different (weighted mean difference of 3.76, 95% CI [(−2.17–9.69)]). Other maternal conditions evaluated were not associated with a higher risk for sPTB.

**Table 2:** Unadjusted risks for sPTB according to some maternal medical history and habits.

Characteristics	sPTB	Controls	RR (95%CI)
**Smoking**			
No smoking	73 (93.6%)	962 (92.5%)	Ref.
Ceased during pregnancy or smoker	5 (6.4%)	78 (7.5%)	0.85 [0.23–3.23]
**Alcohol drinking**^**a**^			
No alcohol	56 (83.6%)	757 (83.5%)	Ref.
Ceased before 1^st^ visit	8 (11.9%)	142 (15.7%)	0.77 [0.34–1.75]
Current drinker at 1^st^ visit	3 (4.5%)	8 (0.9%)	3.96 [1.04–15.05]
**Other Drugs**^**b**^			
Never	61 (95.3%)	839 (94.6%)	Ref.
Ceased before 1^st^ visit	1 (1.6%)	39 (4.4%)	0.37 [0.03–5.18]
Current user at 1^st^ visit	2 (3.1%)	9 (1.0%)	2.68 [0.86–8.33]
**Previous maternal conditions**			
Yes	15 (19.2%)	130 (12.5%)	1.60 [0.89–2.86]
No	63 (80.8%)	910 (87.5%)	Ref.
**Previous abortion**			
Yes	9 (11.5%)	117 (11.3%)	1.03 [0.33–3.19]
No	69 (88.5%)	923 (88.7%)	Ref.
**Mother’s History of PTB**^**c**^			
Yes	8 (11.0%)	98 (9.9%)	1.11 [0.24–5.18]
No	65 (89.0%)	890 (90.1%)	Ref.
**Mother’s History of LBW**^**d**^			
Yes5	5 (7.2%)	111 (11.7%)	0.61 [0.17–2.20]
No	64 (92.8%)	839 (88.3%)	Ref.
**Sister’s History of PTB**^**e**^			
Yes	4 (12.5%)	22 (7.3%)	1.69 [0.52–5.48]
No	28 (87.5%)	279 (92.7%)	Ref.
**Sister’s History of LBW**^**e**^			
Yes	4 (12.5%)	32 (10.6%)	1.18 [0.16–8.91]
No	28 (87.5%)	269 (89.4%)	Ref.
**Total**	**78**	**1,040**	

Missing information for: ^a^144; ^b^167; ^c^57; ^d^99; ^e^17.

LBW: low birth weight; PTB: preterm birth. Values in bold mean they are statistically significant.

**Table 3:** Unadjusted risks for sPTB according to some maternal medical conditions during pregnancy.

Characteristics	sPTB	Controls	RR [95%CI]
Body Mass Index* at first visit (19–21weeks)^**a**^			
Underweight (<21.5 kg/m^2^)	14 (17.9%)	181 (17.4%)	0.99 [0.28–3.55]
Normal weight (21.5–26.2 kg/m^2^)	32 (41.0%)	410 (39.5%)	Ref.
Overweight (26.3–30.9 kg/m^2^)	21 (26.9%)	270 (26.0%)	1.00 [0.54–1.85]
Obesity (>30.9 kg/m^2^)	11 (14.2%)	178 (17.1%)	0.80 [0.52–1.23]
**Quartile of weight gain rate per week (kg/week)**^**b**^			
≤Q1 (≤0.33)	15 (27.3%)	211 (24.9%)	1.01 [0.47–2.20]
Q1-Q2 (0.34–0.49)	14 (25.5%)	200 (23.5%)	Ref.
Q2-Q3 (0.50–0.66)	15 (27.3%)	216 (25.5%)	0.99 [0.43–2.30]
≥Q3 (≥0.67)	11 (20.1%)	221 (26.1%)	0.72 [0.25–2.08]
**Percentile of weight gain rate per week (kg/weeks)**^**b**^			
<p10 (<0.18)	6 (10.9%)	85 (10.0%)	1.15 [0.60–2.23]
p10-p90 (0.18–0.82)	41 (74.5%)	675 (79.6%)	Ref.
>p90 (>0.82)	8 (14.5%)	88 (10.4%)	1.46 [0.97–2.17]
**Cervical length from 18 to 24 weeks**^**c**^			
Mean ± SD	33.1 ± 9.96	36.9 ± 6.35	3.76 [(−2.17)-(9.69)]^#^
≤25 mm	5 (13.5%)	11 (2.5%)	**4.52 [1.08–19.01]**
>25 mm	32 (86.5%)	431 (97.5%)	Ref.
**Urinary tract infection in the first half of pregnancy**^**d**^			
Yes	8 (13.1%)	198 (25.6%)	0.46 [0.20–1.04]
No	53 (86.9%)	574 (74.4%)	Ref.
**Asymptomatic bacteriuria in the first half of pregnancy**^**e**^			
Yes	2 (3.5%)	69 (9.5%)	0.36 [0.04–3.25]
No	55 (96.5%)	654 (90.5%)	Ref.
**Recurrence of any infection**^**§f**^			
Yes	6 (10.9%)	119 (14.0%)	0.76 [0.27–2.16]
No	49 (89.1%)	730 (86.0%)	Ref.
**Vaginal bleeding in the first half of pregnancy**			
Yes	24 (30.8%)	193 (18.6%)	1.85 [0.89–3.84]
No	54 (69.2%)	847 (81.4%)	Ref.
**Number of days with vaginal bleeding in the first half of pregnancy**			
1–3 days	14 (58.3%)	156 (80.8%)	Ref.
>3 days	10 (41.7%)	37 (19.2%)	2.58 [0.62–10.74]
**Total**	**78**	**1,040**	

*According to Atalah body mass index categories at 19 weeks (Atalah E, Castillo C, Castro R, Aldea A. Rev Med Chil. 1997 Dec;125(12):1429–36) ^§^Women who had any infection before first visit (19–21 weeks) and another any infection between first and second visits (between 19–21 weeks and 27–29 weeks); only calculated for women who attended both visits. Missing information for: ^a^1; ^b^215; ^c^639; ^d^285; ^e^338; ^f^32. ^#^WMD, weighted mean difference [95% CI].

Values in bold mean they are statistically significant.

The incidence of sPTB in groups of women with clinical phenotypes including mixed maternal characteristics is shown in Table 4. Some subgroups of women had a higher incidence of sPTB when compared to the general study population, such as underweight women whose weight gain was below Q1 (14.8%), obese women with weight gain >Q3 (14.3%), women with a short cervical length (31.2%) and women with a short cervix that had vaginal bleeding in the first half of pregnancy (40%). None of the maternal phenotypes such as ethnicity, family income or schooling level showed a higher incidence of sPTB.

**Table 4:** Incidence of spontaneous preterm birth according to some maternal clinical phenotypes.

Maternal clinical phenotypes	Incidence of sPTB	[95% CI]
	n/N (%)	
Underweight on enrolment (<21.5 kg/m^2^)* and Weight gain rate per week <Q1	**4/27 (14.8%)**	[0.0–34.7]
Underweight on enrolment (<21.5 kg/m^2^)* and Weight gain rate per week <Q2	4/52 (7.7%)	[0.0–18.7]
Obesity (>30.9)* and Weight gain rate per week >Q3	**3/21 (14.3%)**	[3.9–24.6]
Overweight or Obese* and Weight gain rate per week >Q3	6/76 (7.9%)	[4.1–11.7]
Obesity (>30.9)* and Weight gain rate per week >Q2	5/54 (9.3%)	[0.8–17.7]
Overweight or Obese* and Weight gain rate per week >Q2	11/165 (6.7%)	[3.6–9.7]
Vaginal bleeding and urinary infection in the first half of pregnancy	3/49 (6.1%)	[0.0–15.1]
Short Cervical Length from 18 to 24 weeks	**5/16 (31.2%)**	[0.0–77.2]
Short Cervical Length from 18 to 24 weeks and vaginal bleeding in the first half of pregnancy	**2/5 (40.0%)**	[0.0–91.6]
Low family income and schooling levels (a)	0/42 (0%)	—
Low family income and schooling levels (b)	14/261 (5.4%)	[2.6–8.1]
White, low family income and schooling levels (a)	0/6 (0%)	—
White, low family income and schooling levels (b)	3/48 (6.3%)	[0.0–12.5]
Non-white, low family income and schooling levels (a)	0/36 (0%)	—
Non-white, low family income and schooling levels (b)	11/213 (5.2%)	[2.0–8.3]
White, high family income and schooling levels (a)	11/169 (6.5%)	[3.4–9.7]
White, high family income and schooling levels (b)	13/198 (6.6%)	[3.9–9.2]
Non-white, high family income and schooling levels (a)	5/77 (6.5%)	[0.0–15.9]
Non-white, high family income and schooling levels (b)	9/129 (7.0%)	[0.0–14.8]
**General population of the study**	**78/1165 (6.7%)**	**[4.7–8.7]**

(a) Low income defined as income up to 3,000 US$. High income when above 12,000 US$. (b) Low income defined as income up to 6,000 US$. High income when above 6,000 US$. *(Atalah E, Castillo C, Castro R, Aldea A. Rev Med Chil. 1997 Dec;125(12):1429–36).

Cervical length below 25 mm measured between 18 and 24 weeks of gestation was the only factor independently associated with sPTB on multivariate analysis (adjusted RR_adj_ 4.52, 95% CI [1.08–19.01]) (Table 5). Ethnicity and more than 3 days of vaginal bleeding in the first half of pregnancy were the only significantly maternal characteristics which differs among groups of women with distinct cervical length (≤25 mm, 26–25 mm and >35 mm) (Supplementary S4). The proportion of white ethnicity and more than 3 days of vaginal bleeding in women with a cervix >35 mm and <25 mm were 47.7% and 36.8%, and 5.8% and 0%, respectively.

**Table 5:** Factors independently associated with sPTB: multivariate analyses by non-conditional logistic regression.

Variables	RR_adj_	95% CI	p-value
Cervical Length from 18 to 24 weeks <25 mm*	4.52	1.08–19.01	0.043

Variables included in the model: age (years); marital status; alcohol drinking; previous maternal conditions; initial BMI; Cervical length (<25 mm); vaginal bleeding in the first half of pregnancy; urinary tract infection in the first half of pregnancy; weight gain rate per week (kg) 20–27 weeks >p90; Preeclampsia. *Cervical length: Coefficient: 1.51, Standard error: 0.517; Constant: Coefficient: −2.67, Standard error: 0.138.

## Discussion

### Main findings

The Preterm SAMBA multicentre cohort study found an overall PTB rate of 10.7%, where sPTB accounted for 62.4% of PTB cases (6.7% of all births). In addition, a short cervix at mid-pregnancy was highly associated with sPTB and underweight women whose weight gain was below Q1, obese women with weight gain >Q3, women with a short cervical length, and women with a short cervix that had vaginal bleeding in the first half of pregnancy had a higher incidence of sPTB compared to the overall population.

### Strengths and limitations

We conducted a prospective multicentre cohort study of nulliparous women from five obstetric maternities in Brazil, representing a multi-regional and mixed population in an upper-middle income country. Our study has some limitations: 1) the initial/pre-pregnancy weight was not recorded; 2) transvaginal ultrasound was recorded as ultrasound report or prenatal chart and not all participating women were offered the exam. In addition to these limitations, our study represents local protocols and the reality of obstetric referral centres. Furthermore, a larger sample is presumed to confer more power to the study and other potential risk factors could be identified as significant.

### Interpretations

The PTB rate was slightly lower in our study than in the general female Brazilian population. According to the EMIP study, a multicenter cross-sectional study that provided surveillance for over 33,700 births from 2011 to 2012 in 20 referral Brazilian maternity hospitals, the overall prevalence of PTB was 12.3%^4^; sPTB was responsible for 65% of all PTB. The 2016 official data from SINASC, the national live birth information system, shows a PTB rate of 11.3%^23^. Unfortunately, Brazilian official data does not distinguish PTB rates according to subtypes. A considerable proportion of women in our study were overweight or obese (291/1,165 − 41%) on the first visit (19–21 weeks), had previous chronic medical conditions including anemia, depression or chronic hypertension (145/1,165 − 12.4%), had an annual family income lower than 12,000 US$ (654/1,165 − 56.1%) and had less than 12 years of schooling (785/1,165–65.0%). Considering the characteristics of our population, applying the concept of “low-risk” pregnancy here may be controversial. The sampled population reflects the Brazilian population and is no different from another multicentre study in Brazil^4^. In addition, we focused on nulliparous women to avoid confounders related to a previous history of preterm birth, because it is applicable to every woman (at least once).

A short cervix, defined as a cervical length below 25 mm on second-trimester transvaginal ultrasound, is a significant risk factor for preterm birth. Since the 90 s, a growing body of evidence supports its association with PTB^10,24–26^. Cervical length of nulliparous women seems to be statistically, but not clinically, different from cervical lengths of multiparous women. Iams *et al*. evaluating almost 3,000 North-American women, showed that median cervical lengths were 34.0 mm and 36.1 mm, respectively^10^. More recently, van der Ven *et al*. investigating over 11,000 Dutch women, reported that mean cervical lengths of nulliparous and multiparous were 43.1 mm and 45.1 mm, respectively^12^. In our study, the mean cervical length was 33.1 mm and 36.9 mm in women who had sPTB and term birth, respectively. Cervical length may not clinically differ according to parity, but this seems to vary widely among the population studied. We established 25 mm as a cut-off point for women potentially at higher risk for sPTB. However, the distribution of cervical length in Brazilian women, including the 10^th^ percentile, remains undetermined. It is required for clarification of population characteristics and related adaptations to identify women at higher risk for sPTB.

Recently, a group of experts proposed a new conceptual framework for the study of preterm birth, using maternal clinical phenotypes^27^. A clinical phenotype is characterized by a group of common clinical characteristics observed during pregnancy that is potentially associated with an outcome. Rather than separate women by outcome, the clinical phenotype intends to address the incidence and associated adverse outcomes in women who have common characteristics during pregnancy. It may improve recognition of groups at higher risk for adverse outcomes and enable implementation of targeted interventions for each specific group. We found four maternal phenotypes with at least double the incidence of sPTb compared to the overall population. These phenotypes were based on maternal BMI, weight gain from 20–27 weeks of gestation, cervical length, and vaginal bleeding in the first half of pregnancy. All these characteristics can be easily identified during pregnancy, especially in referral obstetric units where well-trained specialists perform transvaginal ultrasound. A standard threshold for weight gain during pregnancy, dependent on the initial or pre-pregnancy BMI, was not used in this study, since it was not available. More importantly, existing literature supports our findings;obese women with higher weight gain and underweight women with lower weight gain are at increased risk for sPTB^28^. Although each clinical phenotype was composed of a low number of women, resulting in a wide confidence interval of incidence rates, these results are still useful for indicating groups of women that may benefit from further study and are also considered at higher risk for sPTB.

## Conclusion

Our study reinforces that spontaneous preterm birth is a common pregnancy complication in nulliparous women and that cervical length is a remarkable biophysical risk factor. However, instead of routine screening for low-risk nulliparous women, we first suggest a better investigation of the benefits of preventive interventions for this population with a short cervix in the second trimester. In addition, subgroups that have a higher incidence of sPTB should also be further evaluated to find associated factors, perinatal related outcomes, and preventive strategies.

## Supplementary information

Preterm SAMBA Flowchart – Spontaneous preterm birth analysis.

## Data Availability

All relevant data are within the paper, and the authors can make available materials, data and associated protocols if requested.
