# Supplementary material for: Clinical and epidemiological factors associated with spontaneous preterm birth: a multicentre cohort of low risk nulliparous women
Source: Sci Rep. 2020 Jan 21;10:855. doi: 10.1038/s41598-020-57810-4 (PMC6972868; doi:10.1038/s41598-020-57810-4)
Supplement: Supplementary file 1 — Preterm SAMBA Flowchart – Spontaneous preterm birth analysis. [file 41598_2020_57810_MOESM1_ESM.docx]

**Clinical and epidemiological factors associated with spontaneous preterm birth: a multicentre cohort of low risk nulliparous women**

Renato T Souza ^1^, Maria L Costa ^1^, Jussara Mayrink ^1^, Francisco E. Feitosa ^2^, Edilberto A Rocha Filho ^3^, Débora F Leite ^1,3^, Janete Vettorazzi ^4^, Iracema M Calderon ^5^, Maria H Sousa ^6^, Renato Passini Jr ^1^, Philip N Baker ^7^, Louise Kenny ^8^, Jose G. Cecatti ^1^, for the Preterm SAMBA study group

**Table S1. Incidence of preterm birth in the Preterm SAMBA study**

| **Incidence** | **n/N (%)** |
| --- | --- |
| Preterm birth (overall) | 125/1,165 (10.7%) |
| Spontaneous PTB | 78/1,165 (6.7%) |
| Provider-initiated PTB | 47/1,165 (4.0%) |
| **sPTB by Region*** |  |
| Northeast (2 centres) | 13/257 + 21/309 = 34/565 (6.0%) |
| South and Southeast (3 centres) | 12/139 + 14/143 + 18/318 = 44/600 (7.3%) |
| **sPTB categories/severity** | 78 (100%) |
| Late preterm birth (34-36w) | 55 (70.5%) |
| Moderate PTB (32-33w) | 10 (12.8%) |
| Very PTB (28-31w) | 8 (10.3%) |
| Extreme PTB (<28w) | 5 (6.4%) |

*p-value: 0.387

PTB: preterm birth; sPTB: spontaneous preterm birth

**Table S2. Methods for estimating gestational age in the Preterm SAMBA study**

| **Method*** | **sPTB** | **Term birth** |
| --- | --- | --- |
| LMP only | 1 (1.3%) | 15 (1.4%) |
| LMP and US (LMP) | 32 (41.0%) | 449 (43.2%) |
| LMP and US (US) | 21 (26.9%) | 269 (25.9%) |
| US only | 24 (30.8%) | 307 (29.5%) |

*p-value: 0.903

**Table S3. Maternal characteristics according to transvaginal cervical length measured between 18-24 weeks (n=497)**

| **Characteristics** | | **Cervical length ≤25mm** | **Cervical length 26-35mm** | **Cervical length >35mm** | **p-value** |
| --- | --- | --- | --- | --- | --- |
| **Maternal age** **(years)** | |  |  |  | 0.125 |
| ≤19 | | 3 (15.8%) | 53 (26.4%) | 40 (14.4%) |  |
| 20-34 | | 15 (78.9%) | 128 (63.7%) | 208 (75.1%) |  |
| ≥35 | | 1 (5.3%) | 20 (10.0%) | 29 (10.5%) |  |
| **Ethnicity** | |  |  |  | **0.034** |
| White | | 7 (36.8%) | 78 (38.8%) | 132 (47.7%) |  |
| Non-white | | 12 (63.2%) | 123 (61.2%) | 145 (52.3%) |  |
| **Marital status** | |  |  |  | 0.307 |
| With partner | | 16 (84.2%) | 145 (72.1%) | 212 (76.5%) |  |
| Without partner | | 3 (15.8%) | 56 (27.9%) | 65 (23.5%) |  |
| **Maternal Occupation** | |  |  |  | 0.079 |
| Paid work | | 13 (68.4%) | 88 (43.8%) | 166 (59.9%) |  |
| Homemaker | | 4 (21.1%) | 41 (20.4%) | 47 (17.0%) |  |
| Not working | | 2 (10.5%) | 72 (35.8%) | 64 (23.1%) |  |
| **Schooling (years)** | |  |  |  | 0.768 |
| < 12 | | 12 (63.2%) | 127 (63.2%) | 173 (55.4%) |  |
| ≥ 12 | | 7 (36.8%) | 74 (36.8%) | 104 (37.5%) |  |
| **Annual Family Income (US$)** | |  |  |  | 0.295 |
| Up to 3,000 | | 0 (0%) | 3 (1.5%) | 5 (1.8%) |  |
| 3,000 to 12,000 | | 7 (36.8%) | 115 (57.2%) | 122 (44.0%) |  |
| Above 12,000 | | 12 (63.2%) | 83 (41.3%) | 150 (54.2%) |  |
| **Source of prenatal care** | |  |  |  | 0.500 |
| Entirely public | | 16 (84.2%) | 164 (81.6%) | 220 (79.4%) |  |
| Private/insurance/mixed | | 3 (15.8%) | 37 (18.4%) | 57 (20.6%) |  |
| **Smoking** | |  |  |  | 0.316 |
| No smoking | | 17 (89.5%) | 188 (93.5%) | 256 (92.4%) |  |
| Ceased during pregnancy or smoker | | 2 (10.5%) | 13 (6.5%) | 21 (7.6%) |  |
| **Alcohol drinking ^a^** | |  |  |  | 0.571 |
| No alcohol | | 15 (83.3%) | 147 (81.7%) | 189 (79.4%) |  |
| Ceased during pregnancy or drinker | | 3 (16.7%) | 33 (18.3%) | 49 (20.6%) |  |
| **Other Drugs** ^b^ | |  |  |  | 0.109 |
| Never | | 16 (100%) | 176 (95.7%) | 245 (93.9%) |  |
| Ceased during pregnancy or user | | 0 (0%) | 8 (4.3%) | 16 (6.1%) |  |
| **Previous maternal conditions** | | 2 (10.5%) | 40 (19.9%) | 41 (14.8%) | 0.382 |
| **Previous abortion** | | 6 (31.6%) | 31 (15.4%) | 33 (11.9%) | 0.142 |
| **Mother’s History of PTB 29** | | 1 (6.2%) | 24 (12.9%) | 38 (14.3%) | 0.379 |
| **Mother’s History of LBW ^c^** | | 2 (13.3%) | 22 (12.6%) | 36 (14.1%) | 0.814 |
| **Sister’s History of PTB** | | 5 (100%) | 63 (90%) | 95 (88%) | 0.502 |
| **Sister’s History of LBW** | | 5 (100%) | 58 (82.9%) | 91 (89.8%) | 0.305 |
| **Body Mass Index on enrolment** ^d^ | |  |  |  | 0.563 |
| Underweight (<21.5kg/m2) | | 3 (15.8%) | 35 (17.4%) | 37 (13.4%) |  |
| Normal weight (21.5-26.2) | | 6 (31.6%) | 83 (41.3%) | 118 (42.6%) |  |
| Overweight (26.3-30.9) | | 6 (31.6%) | 56 (27.9%) | 69 (24.9%) |  |
| Obesity (>30.9) | | 4 (21.0%) | 27 (13.4%) | 53 (19.1%) |  |
| **Quartiles of weight gain rate per week (kg) 20-27 weeks** ^d^ | |  |  |  | 0.405 |
| ≤0.33 (≤Q1) | | 5 (33.3%) | 28 (15.3%) | 57 (23.3%) |  |
| 0.34-0.49 (Q1-Q2) | | 4 (26.7%) | 56 (30.8%) | 60 (24.5%) |  |
| 0.50-0.66 (Q2-Q3) | | 3 (20.0%) | 58 (31.9%) | 70 (28.6%) |  |
| ≥0.67 (≥Q3) | | 3 (20.0%) | 40 (22.0%) | 58 (23.7%) |  |
| **Percentiles of weight gain rate per week (kg) 20-27 weeks** | |  |  |  | 0.522 |
| <p10 (<0.18) | | 2 (13.3%) | 12 (6.6%) | 23 (9.4%) |  |
| p10-p90 (0.18-0.82) | | 11 (73.4%) | 151 (83.0%) | 207 (84.5%) |  |
| >p90 (>0.82) | | 2 (13.3%) | 19 (10.4%) | 15 (6.1%) |  |
| **Urinary infection in first the half of pregnancy ^e^** | | 2 (14.3%) | 45 (30%) | 50 (23.5%) | 0.203 |
| **Asymptomatic Bacteriuria in the first half of pregnancy ^f^** | | 12 (85.7%) | 105 (70%) | 163 (76.5%) | 0.521 |
| **Recurrence of any Infection§** | | 0 (0%) | 17 (12.3%) | 20 (9.8%) | 0.534 |
| **Vaginal bleeding in the first half of pregnancy** | | 6 (31.6%) | 36 (17.9%) | 55 (19.9%) | 0.372 |
| **Number of days with vaginal bleeding in the first half of pregnancy** | |  |  |  | **<0.001** |
| 1-3 | 6 (100%) | 31 (86.1%) | 39 (70.9%) |  |  |
| >3 | 0 (0%) | 5 (13.9%) | 16 (29.1%) |  |  |
| **Total** | **19** | **201** | **277** | 497 |  |

Missing information for: a) 61; b) 36; c) 51; d) 55; e) 120; f) 142.
